# Preservation of dendritic D2 receptor transmission in substantia nigra dopamine neurons with age

**DOI:** 10.1038/s41598-023-28174-2

**Published:** 2023-01-19

**Authors:** Eva Troyano-Rodriguez, Harris E. Blankenship, Kylie Handa, Sarah Y. Branch, Michael J. Beckstead

**Affiliations:** 1 Aging and Metabolism Research Program, Oklahoma Medical Research Foundation, Oklahoma City, OK USA; 2 Department of Cellular and Integrative Physiology, University of Texas Health, San Antonio, TX USA; 3 Oklahoma City VA Medical Center, Oklahoma City, OK USA

**Keywords:** Neural ageing, Neuronal physiology, Inhibition

## Abstract

Substantia nigra pars compacta (SNc) dopamine neurons are required for voluntary movement and reward learning, and advanced age is associated with motor and cognitive decline. In the midbrain, D2-type dopamine receptors located at dendrodendritic synapses between dopamine neurons control cell firing through G protein-activated potassium (GIRK) channels. We previously showed that aging disrupts dopamine neuron pacemaker firing in mice, but only in males. Here we show that the amplitude of D2-receptor inhibitory postsynaptic currents (D2-IPSCs) are moderately reduced in aged male mice. Local application of dopamine revealed a reduction in the amplitude of the D2-receptor currents in old males compared to young, pointing to a postsynaptic mechanism. Further experiments indicated that reduced D2 receptor signaling was not due to a general reduction in GIRK channel currents or degeneration of the dendritic arbor. Kinetic analysis showed no differences in D2-IPSC shape in old versus young mice or between sexes. Potentiation of D2-IPSCs by corticotropin releasing factor (CRF) was also not affected by age, indicating preservation of one mechanism of plasticity. These findings have implications for understanding dopamine transmission in aging, and reduced D2 receptor inhibition could contribute to increased susceptibility of males to SNc dopamine neuron degeneration in Parkinson’s disease.

## Introduction

Dopamine neurons of the substantia nigra pars compacta (SNc) are integral to motor function and reward learning^1–3^. Dopaminergic behaviors decline with advancing age, with movement deficits, apathy, and depression commonly observed in the elderly population^4,5^. Moreover, aging is the single largest risk factor for development of Parkinson’s disease (PD)^6^; a disease that features motor symptomatology as a consequence of degeneration of SNc dopamine neurons^7^. While a modest level of dopamine cell death is thought to occur with normal age^8–11^, it is insufficient to explain the ~ 60% loss in extracellular dopamine that has been reported in the striatum^12^, suggesting a contribution from declining physiological and/or morphological factors in single neurons. Indeed, we have previously reported that aging affects pacemaker firing frequency and regularity in dopamine neurons from male mice^13^, while firing in female mice is largely preserved^14^. Additionally, we have observed a striking decrease in nimodipine-sensitive (presumably L-type) calcium channel currents in dopamine neurons from aged males, while currents through hyperpolarization-activated cyclic nucleotide-gated (HCN) channels and small-conductance calcium-activated potassium (SK) channels are unaltered. It is less clear how age affects other channels and receptors, and no study to date has explored individual synaptic inputs to dopamine neurons across the lifespan.

Dopamine neurons in the midbrain communicate with each other via dendrodendritic synapses^15^, where D2 type autoreceptors are able to sense the local release of dopamine^16^. Evoking dopamine release through electrical or optogenetic stimulation produces a D2 receptor-mediated inhibitory postsynaptic current (D2-IPSC) that is action potential- and calcium-dependent^17,18^. This form of dendritic neurotransmission proceeds through D2 receptor activation of G_i/o_ proteins and type 2 inhibitory G-protein-activated inwardly rectifying potassium (GIRK) channels^17,19,20^. The resulting inhibitory conductance hyperpolarizes the cell and strongly reduces firing rate^21^. D2 receptor signaling in the midbrain is susceptible to multiple forms of short- and long-term plasticity and is regulated locally by neuropeptides and neuromodulators^18,22–27^. For example, the stress related-molecule corticotropin releasing factor (CRF) transiently increases the amplitude of both D2- and GABA_B_ receptor-mediated currents in a postsynaptic manner consistent with activity at the level of the GIRK channel^23^. The effects of advanced age on D2-IPSCs have not been systematically studied, however humans exhibit a decline in D2-like receptor availability at varying rates depending on the brain region^28–30^, with perhaps no decline at all in the SNc^31^.

The aim of the present work was to delineate the extent to which pre- and postsynaptic components of dopamine neurotransmission in the SNc are affected by advancing age using whole cell patch clamp recordings in mouse brain slices. We hypothesized that dopamine signaling would be reduced both pre- and postsynaptically resulting in smaller and slower currents, and that this deficit in dopamine transmission could affect dopamine cell function in advanced age. We did observe a small but significant reduction in D2 receptor currents in aged males that was postsynaptic and at the level of the D2 receptor. However, both the kinetics of D2-IPSCs and CRF-induced plasticity were preserved with advanced age, as was dendritic branching of individual dopamine neurons. These results indicate that most aspects of dendritic dopamine neurotransmission are remarkably resilient to normal aging.

## Results

### D2 receptor inhibitory postsynaptic currents are decreased in aged male mice

**Figure 1:**
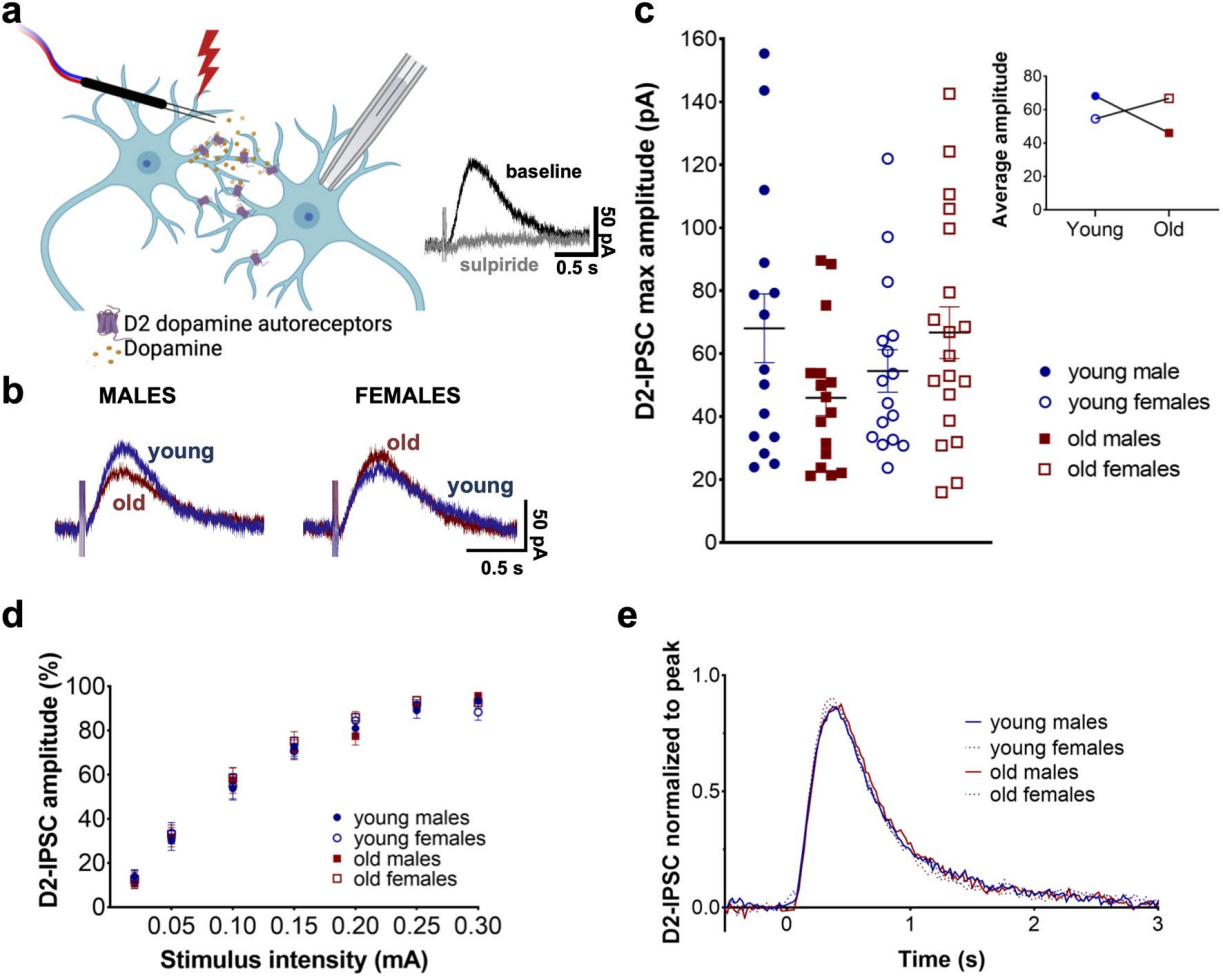
Effect of age and sex on D2 receptor inhibitory postsynaptic currents. (**a**) Schematic diagram of stimulation of sulpiride-sensitive D2-IPSCs. (**b**) Representative traces of D2-IPSCs from old versus young males and females. (**c**) Maximum amplitude of D2-IPSCs for each individual cell for all groups. Inset shows means for all groups with a significant sex x age interaction (*p* = 0.0383). (**d**) Normalized stimulus–response curves show no differences between groups. (**e**) Kinetic analysis of D2-IPSCs amplitudes normalized to maximum outward current show no effect of age or sex. Diagram was created with BioRender.

We acutely prepared horizontal midbrain slices containing the SNc from young (2–8 month) and old (20–25 month) C57Bl/6N mice of both sexes. We then performed whole cell voltage clamp electrophysiological recordings of SNc dopamine neurons (holding voltage −55 mV). In the presence of synaptic blockers, we next used trains of five electrical stimuli to elicit D2-IPSCs (Fig. 1a) over a predetermined range of stimulus intensities (0.02–0.30 mA). Generally, D2-IPSCs appeared to be similar in amplitude and kinetics across ages and sex (Fig. 1b). On average, cells from old males exhibited the lowest maximal amplitudes [Fig. 1c; young males: 68.07 ± 10.89 pA (n = 15); old males: 45.97 ± 5.64 (n = 16); young females: 54.50 ± 6.76 (n = 16); old females: 66.68 ± 8.21 (n = 19)]. A two-way ANOVA on maximal amplitudes did not indicate main effects of sex or age (sex, F_1,62_ = 0.195, *p* = 0.661; age, F_1,62_ = 0.375, *p* = 0.543). We did observe a significant age-sex interaction (F_1,62_ = 4.48, *p* = 0.0383; Fig. 1c inset), however a posthoc analysis did not reveal any significant differences between individual groups (*p* > 0.05; Holm-Šídák's multiple comparisons test). We then normalized each cell to its maximum amplitude, as a difference in the shape of stimulus–response curves could indicate alterations in presynaptic sensitivity or dopamine release probability. Data indicated an expected increase in amplitude with greater stimulus (Fig. 1d, F_6,356_= 352; *P* < 0.0001), which plateaued at the highest stimulus intensities. However, no difference was observed between experimental groups (F_3,62_ = 0.296; *p* = 0.828), nor was there a group-stimulus interaction (F_18,356_ = 0.463; *p* = 0.972). This suggests that the modest differences in aging were not accompanied by an observable decrease in the stimulus–response relationship across groups, arguing against presynaptic differences. An analysis of the kinetics of the D2-IPSCs indicated that the average normalized traces from each group were essentially superimposed (Fig. 1e) with no significant differences observed in time course (F_3,57_ = 0.108, *p* = 0.955). These results suggest that the amplitude and time course of D2-IPSCs are largely conserved across age and sex, and the subtle difference observed in peak amplitudes cannot be explained by altered IPSC kinetics or a shift in the stimulus–response relationship.

### D2-receptor currents induced by exogenous dopamine are decreased in old male mice

**Figure 2:**
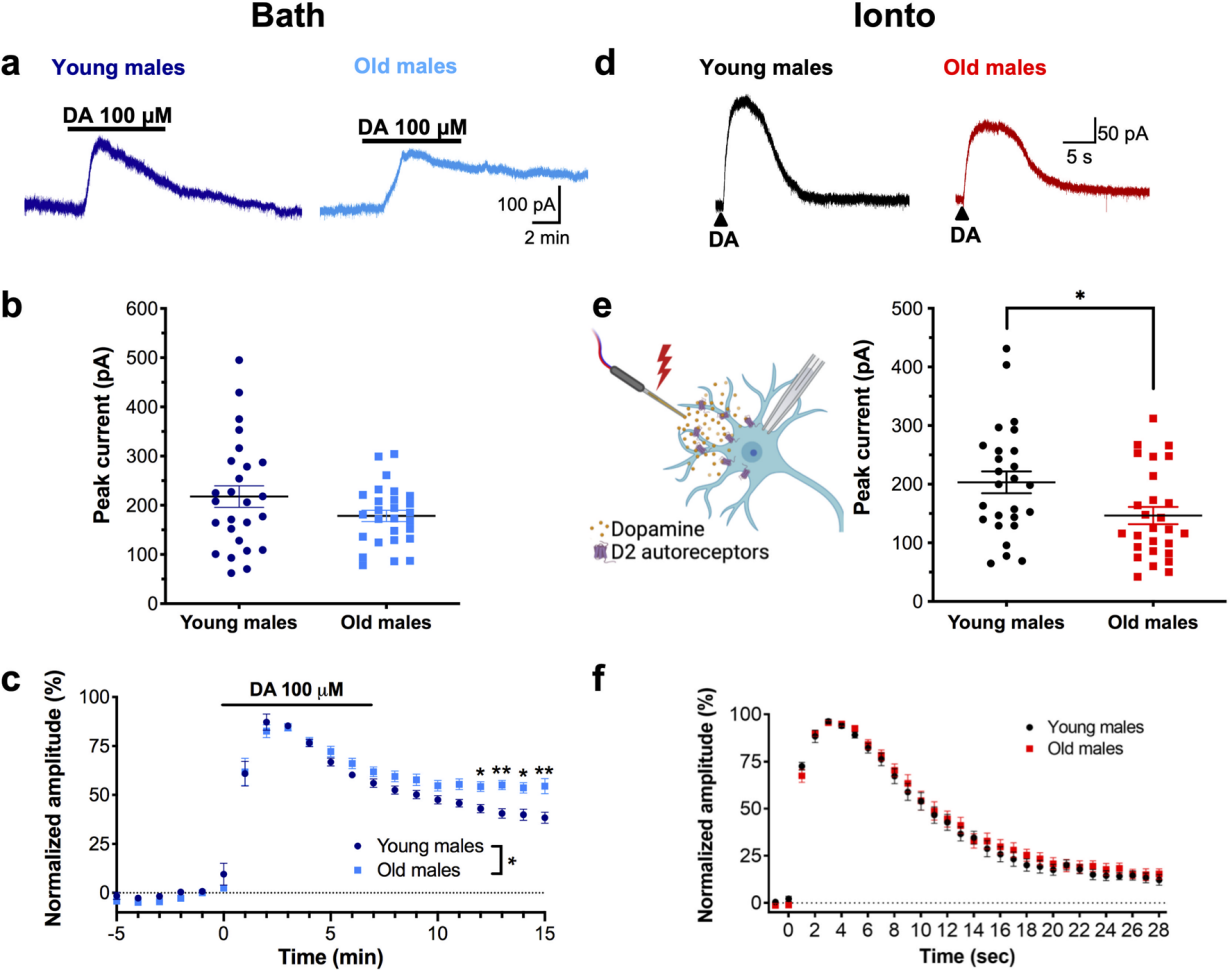
Outward currents in response to exogenous dopamine are reduced in old males. (**a**) Representative traces of D2 receptor mediated currents elicited by bath-application of 100 µM dopamine (DA) for 7 min (horizontal line) from a young (left dark blue trace) and an old mouse (right light blue trace). (**b**) Peak currents from individual cells. Old males tended to have smaller peak currents compared to young males, but this did not reach significance. (**c**) Normalized time course of current after bath application of 100 µM dopamine shows larger responses from old males during washout when compared to young males. (**d**) Representative traces from dopamine currents from young (left trace, black) and old males (right trace, red) when dopamine was applied by iontophoresis (Ionto). (**e**) Diagram depicting iontophoresis of dopamine (left). Peak values from individual cells indicate significantly smaller currents in cells from old males (right). (**f**) Normalized time course of currents elicited by iontophoresis showed no difference in kinetics between old and young males. *p < 0.05. Diagram was created with BioRender.

Since D2-receptor currents tended to be smaller in old males compared to young, we next investigated if this could be due to alterations in postsynaptic D2 signaling in aged males. We voltage clamped dopamine neurons at −55 mV and bath-applied a high concentration of dopamine (100 µM) for 7 min. On average, peak amplitudes were larger in cells from young versus old males [Fig. 2a,b young: 218 ± 21.9 pA (n = 26), old: 178 ± 11.6 pA (n = 27)] but this effect was not statistically significant (unpaired two-tailed t-test, t_51_ = 1.61, *p* = 0.114). Analysis of the normalized time course (Fig. 2c) beginning at the peak with 2-way ANOVA indicated a significant time x age interaction (F_13,540_ = 4.00, *p* < 0.0001) with main effects of time (F_13,540_ = 115, *p* < 0.0001) and age (F_1,45_ = 6.76, *p* = 0.0126). Posthoc analysis revealed a significant simple effect of age during washout from prolonged drug application, indicating a possible decrease in the ability to clear large amounts of dopamine from the extracellular space.

Since bath application of dopamine is relatively slow and current amplitude peaks at a time that desensitization is starting to occur, we repeated this experiment with rapid, local activation of dopamine receptors. We used iontophoresis to rapidly apply dopamine (1 M in the pipette, ejection time: 1 s) near the soma as previously described^24,32^. Analysis of the resulting outward current indicated that neurons from old males exhibit smaller currents compared to young male mice (Fig. 2d,e; young: 203 ± 18.6 pA (n = 26) and old: 146 ± 14.7 pA (n = 27); unpaired t-test, t_51_ = 2.40, *p* = 0.0199). When the currents were normalized to their peak amplitude (Fig. 2f), there was no significant age x time interaction (F_25,1225_ = 0.398, *p* = 0.997) that would indicate a difference in kinetics. These results indicate that rapid, local application of dopamine produces smaller amplitude currents in cells from aged males.

### GABA_B_ receptor-GIRK channel signaling is not reduced in aged male mice

**Figure 3:**
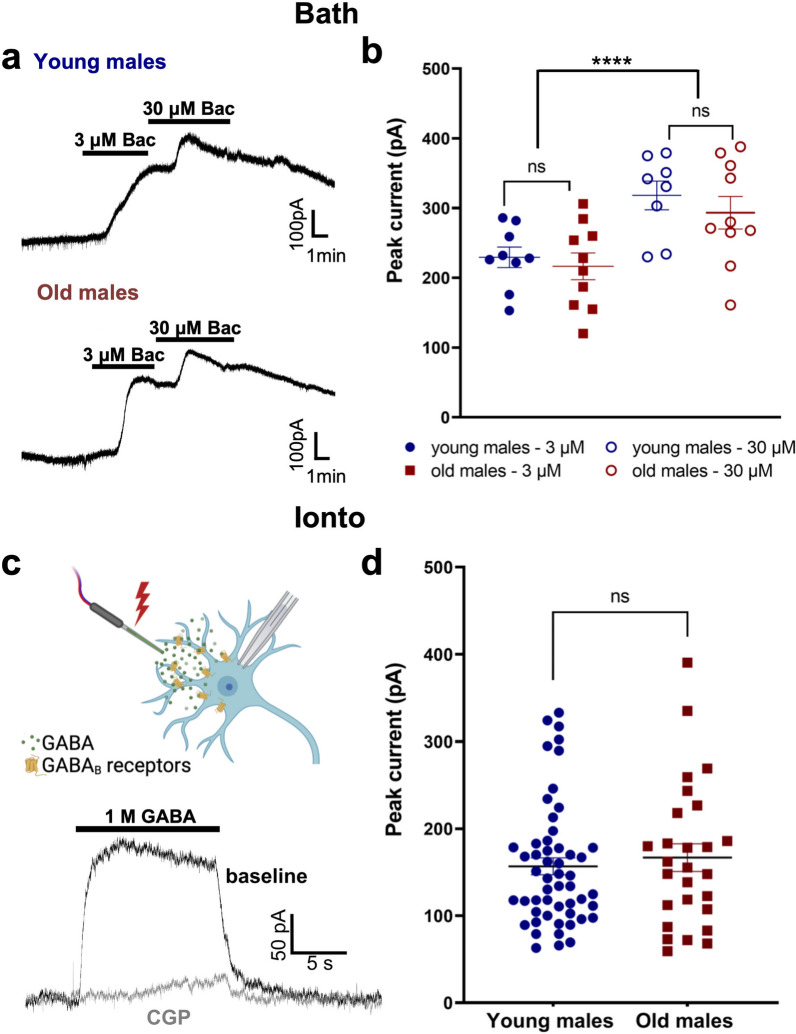
GABA_B_ currents are similar in dopamine neurons from old and young male mice. (**a**) Representative traces of GABA_B_ receptor-mediated currents in young (upper trace) and old (lower trace) males. Two concentrations of the GABA_B_ agonist baclofen (3 and 30 µM) were applied sequentially to activate GIRK channels. (**b**) Maximum amplitudes for individual cells at each concentration showed no effect of age. (**c**) Schematic diagram of iontophoresis (top) and representative traces of GABA_B_ receptor-mediated currents in the presence and absence of 100 nM of the GABA_B_ receptor antagonist CGP 56999a (CGP; bottom). (**d**) Peak individual values of GABA_B_ currents of individual cells. No differences were observed between groups. ****p < 0.0001. Diagram was created with Biorender.

GIRK channels are activated by D2 receptors in dopamine neurons and are responsible for the bulk of the D2-IPSC^17^. We next sought to determine the effect of age on GIRK channel activation mediated by a different receptor. GABA_B_ receptors also activate GIRK channels in dopamine neurons, producing a potassium conductance that is partially overlapping with the D2 receptor-mediated conductance^17,23,33^. We therefore recorded from SNc dopamine neurons in slices from young and old male mice and sequentially bath applied two concentrations of the GABA_B_ receptor agonist baclofen (3 and 30 µM) while in voltage clamp (Fig. 3a). No effects of age were observed in the magnitude of the outward current induced by either concentration of baclofen (3 µM, young: 229 ± 14.7 pA, n = 9; old: 217 ± 19.2 pA, n = 10; 30 µM, young: 318 ± 20.6 pA, n = 8; old: 293 ± 23.3 pA, n = 10; 2-way ANOVA, age: F_1,17_ = 0.492, *p* = 0.493; concentration: F_1,16_ = 110.7, *p* < 0.0001, concentration x age interaction, F_1,16_ = 0.554, *p* = 0.467; Fig. 3b). As with dopamine, we repeated this experiment using local application of GABA by iontophoresis to uncover any effect that could have been masked by early desensitization of the receptor. We used iontophoresis to rapidly apply GABA (1 M in the pipette) in the presence of 100 µM picrotoxin (to block GABA_A_ receptors) and recorded GABA_B_ receptor currents in SNc dopamine neurons from young and old males. Results showed no difference in response between young and old male mice (young: 156.7 ± 9.7 pA, n = 53; old: 166.7 ± 15.8 pA, n = 27); unpaired t-test, t_78_ = 0.569, *p* = 0.571; Fig. 3c,d). This suggests that GIRK channel function is generally preserved in aged male mice and that the differences in D2 currents observed are likely at the level of the D2 receptor.

### D2-IPSC enhancement by the stress neuropeptide CRF is not affected by age

**Figure 4:**
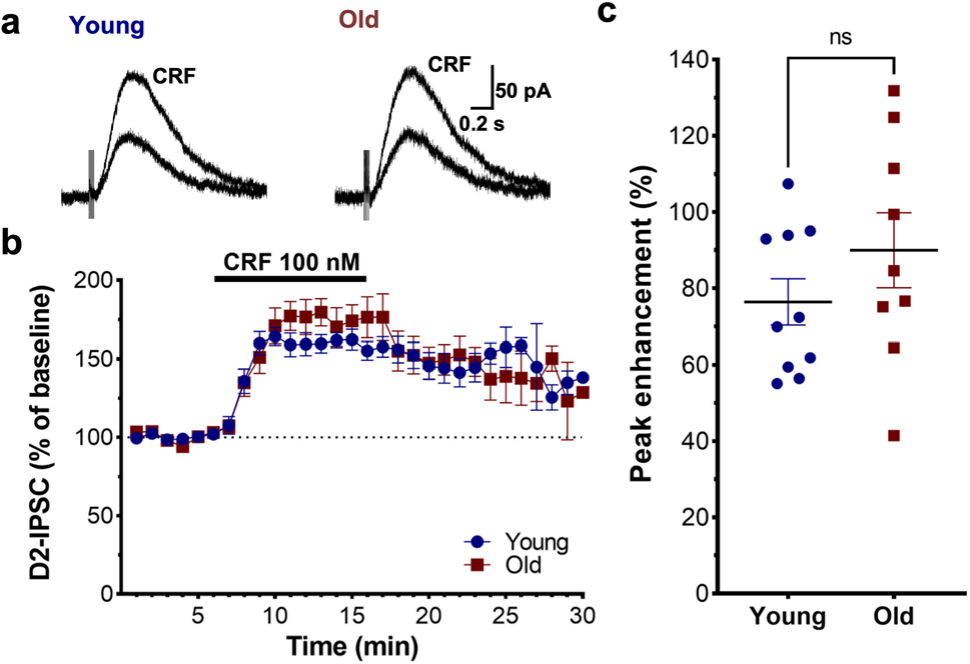
Corticotropin releasing factor (CRF)-induced plasticity is not affected by age. (**a**) Traces from young and old mice depicting representative D2-IPSCs at baseline and after bath application of CRF (100 nM). (**b**) Normalized time course of response to bath-application (horizontal bar) showed no effect of age. (**c**) There was no difference in peak enhancement of D2-IPSCs by CRF in old versus young male mice.

Corticotropin releasing factor (CRF) is a neuropeptide involved in both peripheral and central stress response. Along with its role in initiating the hypothalamic–pituitary–adrenal (HPA) axis loop, CRF is also released in extrahypothalamic sites within the brain^34^. Aging has been associated with decreased responsivity of central and peripheral catecholaminergic systems to acute stress and progressive hypothalamic CRF deficiency in the rat^35,36^. In humans, decreasing CRF concentrations have been observed with aging in the cingulate gyrus (Brodmann areas 23, 24 and 31)^37^. In the ventral tegmental area, CRF-positive axon terminals establish asymmetric (excitatory) synapses on neurons that stain for tyrosine hydroxylase, a dopamine neuron marker^38^. CRF can act through CRF-R1 and CRF-R2 receptors in dopamine neurons^39,40^, and has been reported to alter their firing rate and pattern^41–43^. We have shown that CRF increases D2-IPSCs amplitudes, and this effect is blunted in mice that have been exposed to repeated restraint stress or injections of psychostimulants^23^. Therefore, we next proceeded to determine if CRF-induced modulation of dendritic dopamine signaling is affected by aging. We recorded from SNc dopamine neurons and applied CRF at a concentration that strongly potentiates D2-IPSCs (100 nM for 7–10 min; Fig. 4a). Analysis of the time course (Fig. 4b) and peak enhancement (Fig. 4c) indicated that aging does not alter CRF-induced plasticity of D2 receptor synaptic currents (2-way ANOVA, age: F_1,17_ = 0.169, *p* = 0.687; time: F_29,382_ = 40.3, *p* < 0.0001; time x age interaction F_29,382_ = 1.42, *p* = 0.0765; mean ± SEM, young: 76.4 ± 6.05%, n = 10; old: 90.0 ± 9.83%, n = 9; t_17_ = 1.20, *p* = 0.246). This suggests that this form of D2-IPSC plasticity in dopamine neurons is preserved in aging.

### Somatodendritic morphology is preserved in male mice during aging

**Figure 5:**
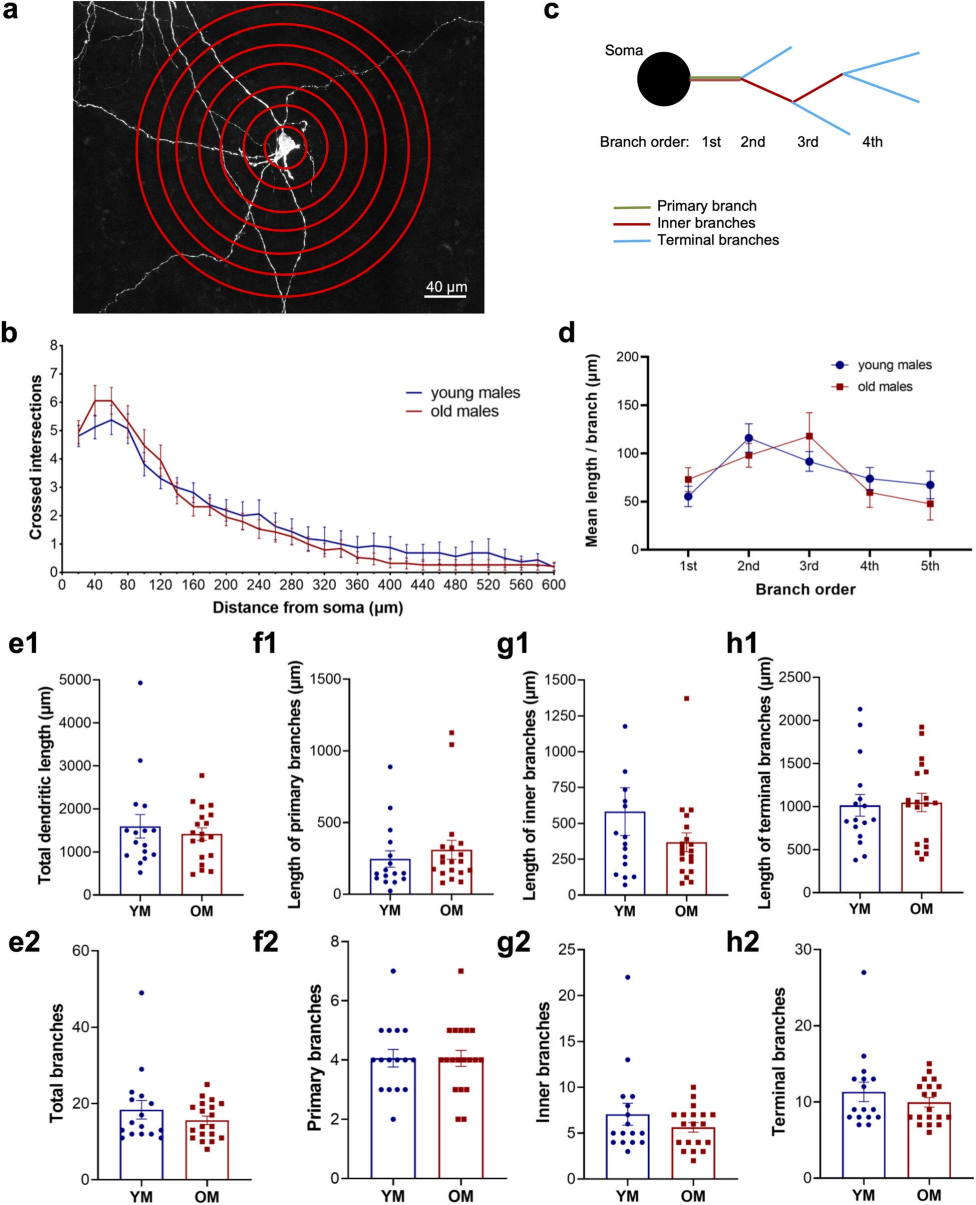
Somatodendritic branching of SNc dopamine neurons is preserved in aged males. (**a**) Representative confocal image of a labelled dopamine neuron with concentric circles at 20 μm increments used for Sholl analysis. (**b**) Average Sholl plot from old and young mice from the soma to 600 μm distance. Analysis was performed from 20 to 400 μm. There was no effect of age. (**c**) Schematic drawing of the soma and dendritic processes used for dendritic measurements. (**d**) Mean length per branch for each branch order from primary branches coming out of the soma to the 5th level, after which the number of branches observed decreased considerably. (**e1-2**) Total length and number of dendrites. Length and number of (**f1-2**) primary, (**g1-2**) inner and (**h1-2**) terminal branches. No differenced were observed in any of the dendritic measurements between young and old males. *YM*  young males, *OM*  old males.

We next sought to determine if differences in somatodendritic morphology could contribute to reduced D2 receptor currents in old males. Altered dendritic morphology has been described in SNc dopamine neurons from adult (5.5–17 months of age) versus juvenile (2–4 weeks) female mice^44^ and by our group in both sexes of MitoPark mice, a genetic animal model of PD^45^. Here, we measured dendritic morphology of male SNc dopamine neurons located along the rostrocaudal and mediolateral axis of the SNc (DV −4.44 to −4.72 mm in the horizontal plane) by filling cells with biocytin included in the recording pipette, conjugating with streptavidin, and performing confocal imaging. Using the Sholl analysis method^46^ we analyzed dendritic arbors between 20 and 400 µm from the soma (Fig. 5a). Dendritic branching was similar from neurons of young and old males (Fig. 5b; 2-way ANOVA, age: F_1,33_ = 0.0131, *p* = 0.910, distance from soma: F_19,627_ = 68.0, *p* < 0.0001; age x distance from soma interaction: F_19,627_ = 1.12, *p* = 0.321).

We further analyzed neurite morphology by quantifying multiple parameters. Neither total neurite length nor number (Fig. 5e1,e2) showed a significant effect of age (length: unpaired two-tailed t-test, t_33_ = 0.610, *p* = 0.546; number: t_33_ = 1.10; *p* = 0.278. Since retraction of dendrites is more likely to happen towards the end of the branches, any subtle shrinkage with age could be reflected in a change of neurite length (or number) at some level of the dendritic arbor (branch order; Fig. 5c). Thus, we took the mean length per branch for the different levels of the dendritic arbor as a key measure of shrinkage. No differences were observed between cells from young and old males (2-way ANOVA, age: F_1,33_ = 0.00251, *p* = 0.960, branch order: F_4,90_ = 4.58, *p* = 0.0021; branch order x age interaction: F_4,90_ = 0.936, *p* = 0.447, Fig. 5d).

Another way to analyze dendritic morphology is by measuring the length and number of primary, inner and terminal branches (Fig. 5c). If somatodendritic morphology is affected by age, we would expect a decrease in the length and number of terminal branches due to inner branches possibly becoming terminal (i.e., primary branches would be less likely to change). No differences were observed in the length or number of primary branches (Fig. 5f1,f2; length: unpaired two-tailed t-test, t_33_ = 0.723, *p* = 0.475; number: t_33_ = 0.0247, *p* = 0.981), inner branches (Fig. 5g1,g2, length: t_33_ = 1.27, *p* = 0.214 ; number: t_33_ = 1.17, *p* = 0.251) or terminal branches (Fig. 5h1,h2, length: t_33_ = 0.203, *p* = 0.840; number: t_33_ = 1.02, *p* = 0.317). We conclude that SNc dopamine neuron morphology is preserved throughout the adult lifespan in males, and that the reduced D2 receptor currents that we observed in old males are not due to shrinkage of the dendritic arbor.

## Discussion

This is the first report of the effects of aging on dopamine D2 receptor transmission in SNc dopamine neurons from mice. D2-IPSC amplitudes were moderately reduced in dopamine neurons from aged males, while the shape of the stimulus–response curve and the IPSC itself were identical across all groups. Iontophoretic application of dopamine proximate to the recorded neuron produced smaller outward currents in aged males, suggesting a postsynaptic decrease in D2 receptor signaling. This reduction did not appear to be caused by impairments at the level of the GIRK channels, as activation of GABA_B_ receptors with baclofen or iontophoresis of GABA did not decline with age. Most other parameters measured were also unchanged by age, including CRF-induced enhancement of D2-IPSCs and the quantification of dendritic arbor morphology of single dopamine neurons. As we have previously noted^13^, perhaps the most striking finding is the number of parameters that were *not* changed by age. However, the fact that many experiments yielded negative results gives us confidence that the observed decrements in D2 currents with age are not due to experimental considerations, such as a phenomenological consequence of the brain slicing procedure.

We previously found that dopamine neuron firing fidelity is only compromised in aged males^14^, and also observed a reduction in the nimodipine-sensitive (presumably L-type) calcium channel conductance with age^13^. Our current results demonstrate that aging can affect synaptic input to dopamine neurons in addition to intrinsic conductances, as evidenced by a reduction in postsynaptic D2 receptor currents. This effect could potentially impact the precision and/or frequency of dopamine neuronal firing, along with other altered ionic conductances or synaptic inputs. We and others have previously shown that synaptically-evoked dopamine release produces a pause in firing^17,47^. We have also long noted a slight but reliable increase in firing rate upon application of the D2 receptor antagonist sulpiride, suggesting modest tonic dopamine inhibition even in brain slices (data not shown). This is consistent with recently published findings from other groups showing an inward current in response to the D2 receptor antagonist sulpiride^48^ and an apparent rightward shift in firing rate histograms in the presence of sulpiride, although the latter was not directly compared^49^. SNc dopamine neurons are notoriously sensitive to insults due to their high energy demands, vast neuritic arbors, large calcium oscillations, and handling of dopamine metabolites, and could be particularly susceptible to oxidative stress^50–52^. Age and sex are the two leading risk factors for development of Parkinson’s disease, with men exhibiting a 1.5–2.0 times higher incidence than women^53^. Increased firing in aged males due to reduced D2 receptor signaling could therefore conceivably interact with other factors to initiate or accelerate processes associated with neurodegeneration due to normal aging and/or diseases such as Parkinson’s.

There are several potential explanations that could explain smaller D2-IPSC amplitudes in old males: (1) decreased presynaptic dopamine release, (2) altered postsynaptic D2 receptor activity, (3) altered GIRK channel activity, or (4) morphological changes, such as reduced dendritic branching or a decrease in synapse number. Although each of these factors does not preclude influences from the others, our findings are most consistent for effects at the level of the postsynaptic D2 receptor. The shapes of the stimulus–response relationship were completely unaffected by age or sex, arguing against presynaptic factors such as reduced release probability. This is also consistent with our results (not shown) using paired trains of stimuli 3 s apart to evoke D2-IPSCs in neurons from male mice which indicated no effect of age (paired-pulse ratio; young: 0.781 ± 0.018, n = 6; old: 0.777 ± 0.011, n = 8; unpaired t-test *p* = 0.863). General G protein/GIRK channel activity was also not obviously affected by age. Responses to bath application of two concentrations of the GABA_B_ agonist baclofen or to GABA iontophoresis did not vary with age, and the kinetics of the D2-IPSC (which could reflect rapid changes in G protein activation or inactivation) were identical across sex and age. We did observe a reduced response to exogenous dopamine in aged males, indicative of reduced D2 receptor responses, although the effect sizes were modest and differences hovered near the edge of statistical significance. Responses to bath applied dopamine seemed to indicate a population of high-responding dopamine neurons in young mice that were not present in old mice, although peak amplitudes exhibited merely a trend toward significance. Interestingly, after several minutes of application, currents observed during washout were significantly increased in aged males. This is not likely due to decreased DAT function, as the kinetics of the D2-IPSC are strongly sensitive to reduced DAT-mediated uptake^17,54^ but were unaffected here. In contrast, peak currents elicited by iontophoretic (rapid) application of exogenous dopamine exhibited a significant reduction in aged males. Although the two data sets looked qualitatively similar, the larger effect of age with local application could be due to the different time course of the respective responses. One limitation in our interpretation is that there are currently no reliable selective antibodies for measuring total or surface expression of endogenous D2 receptors^55^. Regardless, the evidence in hand indicates that the most likely explanation for reduced D2-IPSC amplitudes are due to effects at the level of the postsynaptic D2 receptor. In humans, studies using imaging and/or D2/D3 receptor radioligand tracers indicate variable rates of D2-like receptor decline in normal aging depending on the brain region. Cortical areas seem to show the largest decline in D2-like receptor availability with age (6–16% per decade) while subcortical and striatal regions are relatively spared (1.5–5% per decade)^28–30^. Interestingly, a postmortem study (age 19–88 years) found that D1-like and D2-like receptor density in substantia nigra does not change with age^31^.

An additional factor that could produce smaller postsynaptic D2 currents is impaired somatodendritic morphology in aged males. We previously showed a severely retracted cellular phenotype that worsens with age in the MitoPark mouse model of Parkinson’s disease^45^. However, to our knowledge no previous study has analyzed branching parameters in SNc dopamine neurons across the entire lifespan. Our results here show that somatodendritic morphology of dopamine neurons is preserved in aging throughout several dependent measures including multiple levels of dendritic branching. The reduced D2 currents are therefore apparently dependent on effects of the D2 receptor itself, and not merely a product of smaller cells providing less surface area for postsynaptic D2 receptor expression.

Normal aging is characterized by cognitive impairment that could involve neuronal loss, however many studies have shown minimal cell loss during aging^56,57^. An alternative explanation could be that age produces subtle decrements in morphology of single neurons, and several studies have found differences at the level of dendrites or dendritic spines^58,59^ while others have failed to find differences. For example, Sholl analysis of a three-dimensional reconstruction of corticocortical neurons in macaque monkeys indicated that while dendritic morphology of apical dendrites differed with age, the basal or total arborization remained unchanged^60^. Another study in humans showed that pyramidal neurons in two subdivisions of the hippocampal CA1 region retained complexity in their dendritic arborization in normal aging^61^. Further, a study performed in human prefrontal cortex using Golgi-Cox staining demonstrated that the basal dendritic pattern of pyramidal cortical neurons in layer IIIc (but not in layer V) remained stable during aging^62^. We previously demonstrated that SNc dopamine neurons show no change in cell capacitance with age^13^, which would argue against gross effects on cell surface area or morphology. In this study we focused on just two age groups in adult mice and cannot make conclusions about dendritic morphology at other times. For instance, Rafols et al.^63^ used Golgi staining to show that total dendritic length of aspiny neurons in the striatum gained branching complexity from 3 to 20 months old, but decreased thereafter. Another study in C57BL/6N mice found no differences in dendritic parameters in cortical barrels across the entire lifespan^64^, while a study in the basolateral amygdala of rats showed hypertrophy of the dendritic arbor with aging^65^. Perhaps unsurprisingly, age effects on dendritic arborization exhibit differential patterns based on cell type and brain region, from dendritic morphology not changing at all to growing or regressing early or late in life^66^. Of note, here we did not go beyond 25 months as we did not want to bias the data toward a long-lived population of mice.

We also explored age effects on CRF-induced enhancement of D2 receptor transmission. In male rats, CRF mRNA levels decline with age in the paraventricular nucleus of the hypothalamus, and aging induces greater ACTH and corticosterone responses following a CRF challenge, consistent with hypothalamic CRF deficiency^35^. Other rodent studies also report reduced negative feedback of the HPA axis with aging in response to elevation of glucocorticoids, resulting in a pattern of prolonged activation in response to stress^67^. In humans, reports of age-related changes in HPA axis function are conflicting^68^, and little is known about age-related alterations in CRF function in the SNc in humans or rodents. D2-IPSCs are subject to multiple forms of pre-^22^ and postsynaptic plasticity including long-term depression induced by the peptide neurotensin^18,24,25^ and a transient enhancement produced by CRF application^23^. The CRF enhancement occurs postsynaptically through activation of CRF-R1 and apparently occurs at the level of the GIRK channel, as GABA_B_ signaling is also potentiated. Further, either repeated exposure to psychostimulants (cocaine or methamphetamine) or restraint stress decreases the magnitude of the CRF response^23^. Here we show that aging does not affect enhancement of D2-IPSCs produced by bath-application of CRF. Deficits in synaptic plasticity (long-term potentiation [LTP] or depression of glutamate signaling) have been described with aging that could in part explain cognitive deficits characteristic of advance age^69,70^. For example, extracellular recordings demonstrate that LTP from the perforant path to the dentate gyrus declines more rapidly in 28–34 month-old rats and synaptic enhancement correlates with the ability to perform a circular platform task^71^. Norris and colleagues^72^ reported increased long-term depression in 20–24 month-old rats following low frequency stimulation of CA3 fibers to CA1. In this same work, low frequency stimulation was able to reverse LTP in aged but not in adult animals. Others have proposed that deficits in synaptic plasticity appear early in brain aging^70^. LTP is impaired in 7–10 month old rats when stimulating the Schaffer-commissural projections from a region of hippocampal CA3 that projects only to the basal dendrites of CA1^73^. While it is likely that brain areas exhibit differential sensitivity of synaptic plasticity to advancing age, we have provided evidence that a neuropeptide (CRF)-induced enhancement of D2-IPSCs and a presynaptic short-term depression (indicated by paired pulse ratios) in dopamine neurons are not affected by age.

In summary, our results indicate a modest reduction of postsynaptic D2 receptor current amplitudes in SNc dopamine neurons from aged males. Most of the other parameters measured were unaffected by age, including IPSC shape, indicators of presynaptic dopamine release, plasticity induced by CRF, GABA_B_ receptor signaling, and multiple measures of somatodendritic morphology. The overall conclusion is that dendrodendritic transmission in the SNc is largely preserved with age.

## Materials and methods

### Animals

Male and female C57BL/6N mice aged 2–8 months (“young”) and 20–25 months (“old”) were used in this study. Mice were provided by the National Institute on Aging (NIA) aged rodent colony, and these mice or their first and second-generation offspring were used for all experiments. All mice were group-housed in ventilated standard cages with ad libitum access to food and water. Animal rooms were maintained on a reversed 12-h light/dark cycle (lights off at 9:00 AM) with the temperature held at 26 °C. Procedures were in accordance with the Guide for the Care and Use of Laboratory Animals and approved by the Institutional Animal Care and Use Committee at the Oklahoma Medical Research Foundation (OMRF). Studies were conducted in compliance with ARRIVE guidelines.

### Ex vivo electrophysiology

On the day of the experiment, mice were anesthetized with 2,2,2-tribromoethanol (0.25 g/kg i.p.) and transcardially perfused with ice-cold carboxygenated (95% _2_O_2_ and 5% CO_2_) cutting solution for 45 s. The brains were quickly extracted and placed in a cutting solution containing the following (in mM): 2 KCl, 7 MgCl_2_, 0.5 CaCl_2_, 1.2 NaH_2_PO_4_, 26 NaHCO_3_, 11 D-glucose, and 250 sucrose. Horizontal slices (200 µm) containing the ventral midbrain were obtained using a vibrating microtome (Leica VT1200S). Slices were incubated for 30 min at 33–34 °C with carboxygenated artificial cerebrospinal fluid (aCSF) that also contained the NMDA receptor antagonist MK-801 (30 µM). aCSF contained (in mM): 126 NaCl, 2.5 KCl, 1.2 MgCl_2_, 2.4 CaCl_2_, 1.2 NaH_2_PO_4_, 21.4 NaHCO_3_, and 11.1 D-glucose. Slices were left to stabilize at room temperature for at least 30 min.

Slices were then placed in a recording chamber attached to an upright microscope (Nikon Instruments) and maintained at 33–34 °C with aCSF perfused at a rate of approximately 2 ml/min. SNc dopamine neurons were visually identified based on their location in relation to the midline and the medial terminal nucleus of the accessory optic tract. Dopamine neurons from SNc were further identified by their large soma, slow pacemaker firing (< 7 Hz)^74^ with wide extracellular waveforms (> 1.1 ms), their characteristic hyperpolarization-activated inward cation current (*I*_*H*_) of > 100 pA^75^, and ultimately by measuring D2-IPSCs, which have not been reported in other cell types in wild type mice. Recording pipettes (2.0–2.2 MΩ resistance) were constructed from thin wall capillaries (World Precision Instruments) with a PC-10 puller (Narishige International). Whole-cell recordings were obtained using an internal solution containing the following (in mM): 115 K-methylsulfate, 20 NaCl, 1.5 MgCl_2_, 10 HEPES-K, 2 ATP, 0.4 GTP, and 10 BAPTA, pH 7.35–7.40, 269 –272 mOsm.

Electrical stimulation was performed with a bipolar platinum electrode with tip separation of 305 μm, placed in the slice ~ 100 μm caudal to the cell being recorded. The membrane voltage was held at -55 mV and synaptic currents mediated by dopamine could be detected in most of the cells using 5 pulses (0.5 ms duration) applied at 40 Hz once every 60 s. For stimulus–response curve experiments, intensity of the electrical stimulation was applied in an incremental manner (0.02–0.05–0.1–0.15–0.2–0.25–0.3 mA). For experiments using bath applied dopamine, peak values were determined from continuous recordings, while data from prolonged application (Fig. 2c) were collected every 1 min and normalized to that cell’s peak amplitude. Iontophoretic pipettes were pulled from thin-wall microelectrodes (resistance ~ 100 MΩ), filled with dopamine (1 M), and placed ~ 20–40 µm from the cell. A holding current of approximately -3 nA was applied to prevent passive leakage of dopamine, and dopamine was ejected as a cation with a single 200 nA, 1000 ms pulse with an ION-100 single-channel iontophoresis generator (Dagan Corporation). GABA_B_ receptor currents were also evoked by iontophoresis in the presence of the GABA_A_ receptor blocker picrotoxin (100 µM). GABA (1 M in the pipette, pH 4.0) was ejected as a cation with a pulse of 200 nA for 8–12 s. Leakage was prevented by the application of a negative backing current of -5 to -10 nA.

### Drugs

D2 receptor-mediated outward currents were recorded in the presence of the following receptor antagonists (all from Sigma-Aldrich): MK-801 (30 µM added to the recovery bath, NMDA), picrotoxin (100 μM, GABA_A_), CGP 56999a (100 nM, GABA_B_), DNQX (6,7-dinitroquinoxaline-2,3(1H,4H)-dione) (10 μM, AMPA), and hexamethonium (100 μM, nicotinic acetylcholine). For bath application experiments, drug concentrations were as follows: dopamine hydrochloride (Sigma-Aldrich, 100 μM), CRF (Tocris, 100 nM), and baclofen (GABA_B_ agonist, Sigma-Aldrich, 3–30 μM). GABA, Mg ATP, Na GTP, Na HEPES, K HEPES, and BAPTA were obtained from Sigma-Aldrich. K-methyl sulfate was from MP Biomedicals, LLC.

### Dopamine neuron morphology

To analyze somatodendritic morphology of dopamine neurons, biocytin (Sigma) was added to the internal solution at a final concentration of 0.25% and allowed to diffuse within the cytoplasmic compartment for 10–12 min with the series resistance stable at < 10 MΩ. Once cells were filled, and for a maximal preservation of intact soma and dendrites, the glass pipette was slowly retracted horizontally and then vertically until the pipette was outside the slice. A 5–10 min washout with aCSF was allowed to remove any extracellular presence of biocytin molecules that could interfere with the subsequent staining procedure. The 200 µm brain sections were then fixed in 4% paraformaldehyde in phosphate-buffered saline (PBS) for 12 h and washed three times with PBS at room temperature for at least 30 min. All staining was conducted on freely floating sections, and incubations were conducted with gentle agitation at room temperature. Post-fixed tissue sections were blocked with 5% normal donkey serum (NDS) in PBS with 0.2%Tween 20 (PBST) for 1 h. Sections were washed at least three times with PBST and incubated with native Streptavidin protein DyLight 488 (1:750 dilution; Abcam, ab134349) in 0.5% fish skin gelatin with PBST for 2 h. The sections were then subjected to two rinses in PBST followed by two washes in PBS before mounted on gelatin-coated slides, and cover slips were applied with ProLong Gold Antifade Mountant (Thermofisher Scientific, Waltham, MA). Z-stack images were captured using a Zeiss LSM 710 Confocal microscope (Oberkochen, Germany) at 20 × magnification with 1 μm step size using Zen software (black edition). Neurites were traced using ImageJ and the Simple Neurite Tracer (SNT) plugin. Neurite tracings were used to calculate dendritic measurements and arborization patterns via Sholl and SNT analysis.

### Statistics

Unpaired *Student’s* t-tests or one-way ANOVA was performed to analyze differences between two or more groups. Two-way ANOVAs were used to analyze differences in current amplitudes over time between groups, along with Holm-Šídák's multiple comparisons test (Prism 9.3.1, Graphpad). Summary data in graphs is presented as mean ± SEM. Significance was set a priori for all experiments at a *p* value < 0.05.

## Data Availability

Data are available from the corresponding author (MJB) upon request.
